# Expenditure Reductions Associated with a Social Service Referral Program

**DOI:** 10.1089/pop.2017.0199

**Published:** 2018-11-28

**Authors:** Zachary Pruitt, Nnadozie Emechebe, Troy Quast, Pamme Taylor, Kristopher Bryant

**Affiliations:** ^1^ Department of Health Policy and Management, College of Public Health, University of South Florida, Tampa, Florida.; ^2^ Department of Epidemiology and Biostatistics, College of Public Health, University of South Florida, Tampa, Florida.; ^3^ Center for CommUnity Impact, WellCare Health Plans, Inc., Tampa, Florida; ^4^ HCA Healthcare West Florida Division, Tampa, Florida.

**Keywords:** social determinants of health, costs, accountable care, Medicaid, Medicare Advantage

## Abstract

Recent health system innovations provide encouraging evidence that greater coordination of medical and social services can improve health outcomes and reduce health care expenditures. This study evaluated the savings associated with a managed care organization's call center-based social service referral program that aimed to assist participants address their social needs, such as homelessness, transportation barriers, and food insecurity. The program evaluation linked social service referral data with health care claims to analyze expenditures in 2 annual periods, before and after the first social service referral. Secondary data analysis estimated the change in mean expenditures over 2 annual periods using generalized estimating equations regression analysis with the identity link. The study compared the change in mean health care expenditures for the second year for those reporting social needs met versus the group whose needs remained unmet. By comparing the difference between the first and second year mean expenditures for both groups, the study estimated the associated savings of social services, after controlling for group differences. These results showed that the decrease in second year mean expenditures for the group of participants who reported all of their social needs met was $2443 (10%) greater than the decrease in second year mean expenditures for the group who reported none of their social needs met, after controlling for group differences. Organizations that integrate medical and social services may thrive under policy initiatives that require financial accountability for the total well-being of patients.

## Introduction

Population health suffers when weak ties isolate medical care from social services.^1–3^ The United States trails similar countries in the integration of medical care delivery and social services, such as food, transportation, and housing assistance.^4,5^ Vulnerable patients, including older adults and people with low incomes or chronic illnesses, often face urgent nonmedical needs that impact medical care utilization.^6–9^ Recent health system innovations in the United States demonstrate that greater attention to social determinants of health can improve health outcomes and reduce health care expenditures.^10–13^ However, more research is necessary to evaluate the association of medical system coordination of social services with health care expenditures.^11,14,15^

Health*Connections* represents a new model of medical and social service coordination.^16,17^ The program was developed by WellCare Health Plans, Inc., a managed care organization (MCO). Since September 2014, participants with unmet social needs have contacted the call center-based program to obtain free referrals to a nationwide network of local, community-based public assistance programs. The program matches participant needs to available social services.^17^ Similar to 2-1-1 social service information phone lines, the MCO call center representatives refer participants to many different types of social services, including transportation, food programs, financial assistance for utilities, education programs, and housing services.^18,19^

The Health*Connections* program representatives offer 2 additional services beyond the typical 2-1-1 service offerings. First, because of limited resources of 2-1-1 systems, community organizations must add and maintain their information in the various 2-1-1 system online databases.^20^ Health*Connections* employs a team of individuals responsible for identifying, collecting, maintaining, and analyzing the database of community-based social service organizations. Also, the call center is staffed with representatives who have personal experiences with the social service system, an approach that enables identification of the best match of services for participants' unmet social needs.^16^ Program representatives may make referrals to social service organizations for multiple social needs, such as help with utility payments or transportation to the doctor. The program tracks each referral in the tracking database separately. Program representatives follow up with the participants to confirm whether the social services met their social needs.

## Methods

### Study design

This retrospective, secondary data analysis examined the association between met social needs and health care expenditures. The study compared the change in mean health care expenditures for 2 groups of participants – all social needs met versus no social needs met – in the year before the social service referral and year after social service referral.

### Study population

The study sample included participants insured through Medicare Advantage or Medicaid managed care in 14 states who called WellCare's Health*Connections* program between January 1, 2015, and March 1, 2016 seeking referrals to a broad array of community-based public assistance programs, such as housing services and utility assistance. Participants learned of the referral program through health plan materials (eg, provider directories), advertisements, health care service providers, and the MCO customer service and case management units.^16^ The MCO program's database contained hundreds of organizations offering more than 60 categories of social services. All study participants identified at least 1 type of unmet social need and received at least 1 referral to a social service organization. Program representatives provided contact information of the organization to participants, who then were responsible for contacting and utilizing the referred service.

Program representatives followed up with all participants, usually within 2 weeks of the referral, to track whether the participant reported each of their identified social needs as met or not met. For example, if a participant received 3 referrals—1 food bank, 1 utility assistance, and 1 transportation service—and subsequently reported that they had each of the 3 identified social service needs met, then that participant had “all of their needs met.” The group of participants who reported that they had all of their identified needs met were compared to those who reported that none of their identified needs were met.

Participants who reported that only some of the referred services met their identified social needs were excluded in the primary analysis, but included in the sensitivity analysis. Participants were excluded from all analyses if the use of the social service could not be confirmed, if they had incomplete sociodemographic data, or if they were not enrolled continuously with the MCO throughout the 2-year study period.

### Outcome measures

Social service referral tracking data were connected to MCO medical claims for each participant with records between January 1, 2014, and March 1, 2017. The outcome measures were the change in mean health care expenditures from the 12 months before the index referral to 12 months after. The index dates demarcating each 12-month period were defined as the earliest referral date when the social needs were met or the earliest referral date when the social needs were not met.

Mean total health care expenditures for all participants were calculated by summing the fee-for-service expenditures paid by the MCO in unadjusted US dollars 12 months before and after the referral index date. Expenditures were grouped by health service type, including professional (office-based physician, emergency department [ED]-based physician, and laboratory), inpatient, outpatient (hospital-based, skilled nursing facility, ambulatory surgery center, and urgent care clinic), ED (facility only), and prescription drugs.

### Study variables

Following Andersen's Behavioral Model of health services utilization, the study's statistical models controlled for predisposing, need, and enabling factors.^21^ Predisposing characteristics included state of residence (Arkansas, Connecticut, Florida, Georgia, Illinois, Kentucky, Louisiana, Missouri, Mississippi, New Jersey, New York, South Carolina, Tennessee, and Texas), metropolitan status (rural as <250,000 residents or urban as >250,000 residents),^22^ race/ethnicity (black, white, Hispanic, or Native American/Asian/other), age, and sex. To account for illnesses that directly generate the need for health services, a categorization of the count of comorbid diagnoses (none, 1–3, and >3) was included.^23,24^ Enabling variables included participant insurance type (Medicaid managed care or Medicare Advantage) and whether they were actively enrolled in case management during the study period.

### Statistical analyses

Pre-referral characteristics of the study groups are described using means and proportions. The differences in proportions among study groups were tested using a chi-square test and mean differences in age were tested using the 2 independent sample *t* test. The frequency distribution of social service needs met also was examined.

Mean differences in health care expenditures over the 2 annual periods within and between the 2 study groups were estimated. Typical of expenditure measures, the distribution of the means was skewed to the right. Measurement of the mean differences was utilized to determine the relationship of the referral program to total health care costs.^25,26^ To determine the relative change in expenditures over time, generalized estimating equations (GEE) with the identity link were utilized to account for skewness, heteroscedasticity, and within-subject effects over time periods. Components of the GEE model included indicators to represent the study groups, time period, an interaction of the group reporting all needs met with time-period, and predisposing, need, and enabling factors, as already described. The coefficient of the interaction term represented the impact of social services on the mean change in expenditures between study groups. The index date in the model was utilized to neutralize any influence the average index date among groups could have on the study results.

### Additional analysis

A sensitivity analysis was conducted to define the study groups in a different manner. The group who reported at least 1 social need met was compared to the group of participants who reported none of their social needs met. Although the primary analysis enabled evaluation of the program under ideal conditions, the inclusion of participants with partially met needs provides some practical information on the marginal influence of the program.

In addition, analyses of 2 subgroups of the study participants were conducted, including participants identified by the MCO as high risk through a variety of a health risk appraisal (HRA) screening questionnaires. The MCO's HRA algorithms considered individual factors, such as poor functional or nutritional status, to predict future heath care utilization.^27^ The subgroups defined by different insurance types also were examined separately.

All tests were 2-sided and analyses were performed using SAS version 9.4 (SAS Institute, Inc., Cary, NC). The study was classified as exempt by the University of South Florida Institutional Review Board (#Pro00028372) as the program evaluation is based on analysis of data collected previously for existing program operations. In addition, informed consent for the secondary data analysis was not required as all data were de-identified by removing any personally identifiable information.

## Results

Among the 2718 participants in the analysis, 1521 (56%) reported all of their identified social needs were met and 1197 (44%) reported that none of their needs were met. Among the 5035 social services received in the second year, the 10 most commonly reported social services, which represented 78.7% of all services, were medical transportation support (14.5%), utility financial assistance (12.1%), food pantry or program (11.8%), free or reduced vision services (9.9%), general financial assistance (7.9%), free or reduced dental services (6.1%), medication assistance (5.7%), general transportation support (4.2%), housing support (3.8%), and rent assistance (2.7%).

Table 1 presents the descriptive statistics of participant characteristics by study group. There were statistically significant differences across primary analysis study groups for all covariates except for sex and race/ethnicity.

**Table 1.** Pre-Referral Characteristics of Study Population (n = 2718)

*Characteristic*	*All social needs were met (*n* = 1521)*		*No social needs met (*n* = 1197)*		P *value*^b^
**Age**^a^	52.7 ± 17.2		57.6 ± 17.79		<.0001
	n	*%*	n	*%*	
**Sex**					0.97
Female	1024	67.32	805	67.25	
Male	497	32.68	392	32.75	
**Insurance Type**					<0.0001
Medicaid Managed Care	781	51.35	292	24.39	
Medicare Advantage	740	48.65	905	75.61	
**Race**					0.06
Black	179	11.77	172	14.37	
White	1231	80.93	925	77.28	
Hispanic	58	3.81	61	5.10	
Other	53	3.48	39	3.26	
**Metropolitan Status**					<0.0001
Rural	534	35.11	289	24.14	
Urban	987	64.89	908	75.86	
**Identified as High Risk**					<0.0001
No	646	42.47	679	56.73	
Yes	875	57.53	518	43.27	
**Number of Comorbidities**					<0.0001
None	558	36.69	550	45.95	
1 to 3	585	38.46	438	36.59	
>3	378	24.85	209	17.46	
**State of Residence**					<0.0001
Arkansas	68	4.47	116	9.69	
Connecticut	15	0.99	12	1.00	
Florida	110	7.23	110	9.19	
Georgia	271	17.82	287	23.98	
Illinois	52	3.42	52	4.34	
Kentucky	672	44.18	172	14.37	
Louisiana	22	1.45	27	2.26	
Missouri	8	0.53	7	0.58	
Mississippi	134	8.81	178	14.87	
New Jersey	13	0.85	9	0.75	
New York	21	1.38	20	1.67	
South Carolina	6	0.39	13	1.09	
Tennessee	71	4.67	152	12.70	
Texas	58	3.81	42	3.51	

^a^ Represents mean and standard deviation.

^b^ *P* values are from a 2 independent sample *t* test for the difference in mean age and chi-square test for differences in the proportions of other predisposing, need, and enabling factors across study groups.

As shown in Table 2, the group reporting all of their needs met had higher predicted total expenditures than the group of participants who reported not having any of their social needs met in both the first and second years. Expenditures for the group reporting all their needs met in the first year and second years is consistently higher for each of the service types, such as inpatient and ED.

**Table 2.** Estimated Mean Annual Expenditures for the Group Reporting All Needs Met vs the Group Reporting No Needs Met, After Adjusting for Group Differences

	*12 months pre referral*		*12 months post referral*		*Change (post–pre)*			
*Total*	*Mean^a^*	*SE*	*Mean^a^*	*SE*	*Mean difference*	*SE*	*% change*	P *value*
All needs met	$23,553	$990	$20,952	$879	−$2601	$735	−11%	0.0004
Needs were not met	$17,338	$824	$17,180	$901	−$158	$817	−1%	0.86
Between group difference	$6215		$3772		−$2443	$1058	−10%	0.04
**Professional Services**								
All needs met	$4687	$176	$4069	$170	−$617	$175	−13%	0.0004
Needs not met	$3733	$240	$3388	$146	−$345	$228	−9%	0.13
Between group difference	$954		$681		−$273	$288	−4%	0.34
**Inpatient**								
All needs met	$8237	$751	$6018	$620	−$2219	$489	−27%	<.0001
Needs not met	$6287	$497	$5226	$509	−$1061	$641	−17%	0.1
Between group difference	$1950		$792		−$1158	$806	−10%	0.15
**Outpatient**								
All needs met	$3660	$221	$2937	$177	−$723	$203	−20%	0.0004
Needs not met	$2461	$223	$2362	$193	−$99	$227	−4%	0.66
Between group difference	$1199		$575		−$624	$305	−16%	0.04
**Emergency Department**								
All needs met	$1273	$72	$1295	$86	$22	$73	2%	0.77
Needs not met	$815	$71	$843	$89	$28	$65	3%	0.66
Between group difference	$459		$452		−$6	$98	−1%	0.95
**Prescription Drugs**								
All needs met	$5696	$309	$6632	$353	$936	$291	16%	0.001
Needs not met	$4042	$331	$5361	$487	$1319	$418	33%	0.002
Between group difference	$1654		$1271		−$383	$509	−17%	0.45

^a^ Mean and standard error (SE) were obtained from the generalized estimating equation model. Model adjusts for age, sex, race/ethnicity, state of residence, metropolitan status, comorbidity, case management enrollment, and type of health plan.

Participants who reported all their social needs were met experienced an 11% reduction in total health care expenditures in the 12 month post-referral period. The predicted reduction for this group was estimated to be $2601 (SE = $735; *P* < 0.001) in the second year, after controlling for group differences (Table 2). Conversely, among those who reported that their needs were not met, a small and nonsignificant reduction in the second year was observed (*P* = 0.86) (Table 2). When examining the pre–post mean difference between the 2 study groups, the analysis revealed that the group reporting all of their needs met had a greater reduction in mean expenditures in the year following the referral. This reduction in total expenditures represents a comparative 10% reduction (*P* = 0.04) in the second year, after controlling for group differences (Table 2).

Table 2 also itemizes the results by service type. For inpatient services, a 10% reduction in expenditures between study groups was observed in the second year, although of limited significance (*P* = 0.15), compared to participants who reported not having any of their social needs met. For outpatient services, the relative reduction in expenditures between participant groups was 16% (*P* = <0.04). The second year point estimates for professional services were small and nonsignificant, and the prescription drug and ED estimated expenditures increased in the second year for both groups.

The subgroup analysis revealed relative differences in expenditures in the 12 months before and after the index referral date for both insurance type and high-risk stratifications (Table 3). The relative difference for participants insured by Medicare Advantage was 3.3% (β = $589; SE = $1473; *P* = <0.67) and the relative difference for participants insured by Medicaid managed care was −1.4% (*P* = <0.14). Among high-risk participants, the relative difference between groups in the second year was −8.6% (*P* = 0.10). Among those participants not identified as high risk, the relative difference in expenditures was −5.0% (*P* = 0.72) (Table 3).

**Table 3.** Estimated Mean Change in Total Expenditures Across Various Subgroups

*Subgroups*	n	*12 months pre referral Mean*^a^	*12 months post referral Mean*^a^	*Change (post–pre) Mean diff.*	*Percent change (change/pre referral)*	*SE*	P *value*
**Medicare Advantage**							
All needs were met	740	$16,978	$18,652	$1674	9.8%	$950	0.08
No needs were met	905	$16,757	$17,842	$1085	6.5%	$1125	0.33
Between group difference		$221	$810	$589	3.3%	$1473	0.67
**Medicaid Managed Care**							
All needs were met	781	$29,784	$23,130	−$6653	−22.3%	$1093	<0.0001
No needs were met	292	$19,140	$15,128	−$4012	−20.9%	$1435	0.005
Between group difference		$10,644	$8002	−$2641	−1.4%	$1804	0.14
**Identified as High Risk**							
All needs were met	875	$31,050	$26,080	−$4969	−16%	$876	<0.0001
No needs were met	518	$24,702	$22,867	−$1835	−7.4%	$1572	0.24
Between group difference		$6347	$3213	−$3134	−8.6%	$1893	0.10
**Not Identified as High Risk**							
All needs were met	646	$13,400	$14,005	$605	−4.5%	$964	0.53
No needs were met	679	$11,720	$12,842	$1122	−9.5%	$1095	0.31
Between group difference		$1680	$1163	−$516	−5.0%	$1459	0.72

^a^ Mean and standard error (SE) were obtained from the generalized estimating equation model. Model adjusts for age, sex, race/ethnicity, state of residence, metropolitan status, and comorbidity.

The sensitivity analysis shown in Table 4 illustrates the comparison of the mean expenditures for participants with at least 1 met social need compared to those whose social needs were not met. Participants with at least 1 social need met experienced a relative 7% reduction in total health care costs compared to the control group (β = -$1,572, SE = $1137; *P* = 0.17).

**Table 4.** Estimated Mean Annual Total Health Care Costs for Participants with at Least One Social Need Met Compared to Those Whose Needs Were Not Met, After Adjusting for Group Differences (n = 3225)

	*12 months pre referral*		*12 months post referral*		*Change (post–pre)*			
	*Mean*^a^	*SE*	*Mean*^a^	*SE*	*Mean difference*	*% change*	*SE*	P *value*
At least 1 need met	$22,757	$793	$21,027	$757	−$1730	−8%	$666	0.009
Needs not met	$17,338	$824	$17,180	$901	−$158	−1%	$922	0.86
Between group difference	$5419		$3847		−$1572	−7%	$1137	0.17

^a^ Mean and standard error (SE) were obtained from the generalized estimating equation model. Model adjusts for age, sex, race/ethnicity, state of residence, metropolitan status, comorbidity, case management enrollment, and type of health plan.

## Discussion

This program evaluation investigated an MCO's effort to address diverse social needs through integration of medical care and social support. The results showed that participants who reported all their social needs met had higher health care costs than those who reported none of their needs met in the pre-referral year; these costs remained higher even in the post-referral year after all of their social needs were met. Although addressing social needs may not have reduced the costs to the level of those with unmet social needs, the larger decrease in costs represents a significant finding for organizations that accept financial risk for the care of populations of patients. These results reinforce the need for policies that encourage organizations to accept financial responsibility for addressing social determinants of health through nonmedical interventions.^28–30^

In the primary analysis, the mean health care expenditure for each group decreased in the 12 months after the social service referral index date. The reductions in expenditures likely are related to unknown effects outside fulfilling the social service need among these participants. This phenomenon makes the application of the comparison group in this analysis essential. Therefore, this study reports the *difference* in the change in mean second year expenditures between study groups. Although there is a reduction in mean expenses for both groups in the second year, an additional 10% reduction exists for those who reported their social needs met, compared to those who did not. This relative $2443 reduction in the second year may be related to addressing their social needs.

Effective health care delivery may require interventions from a broad array of community-based organizations that act beyond the scope of the medical care provider.^31^ These findings are consistent with a growing body of literature showing that programs that coordinate multiple types of social services may reduce health care expenditures or utilization.^11,12,19,32,33^ Although some programs have targeted specific social determinants of health, such as food insecurity^6,9,34–36^ or housing needs,^7,37,38^ almost half of studies examined in a recent systematic literature review included interventions designed to address multiple unmet social needs.^15^ The wide variation of service types represented in Health*Connections* referral data is consistent with this multi-sector approach.

In addition, these analyses aimed to evaluate the social service referral program as a whole; therefore, the key variable of interest was self-identified social service needs. Health*Connections* referred participants to services with a variety of social service intensity (eg, food bank vs. homeless shelter). Rather than measuring the different types or counts of individual social services, which would wrongly assume that the various services are comparable, this study categorized participants according to whether they reported that services addressed self-identified need. Future analysis seeking to evaluate the relationship of each service type to health care expenditures may be warranted.

These primary results indicate that meeting social needs may lower total health care expenditures through a reduction in a variety of medical care service types. Inpatient cost reductions show the largest point estimate reduction between study groups, although of limited statistical significance. For outpatient services, which include claims for hospital-based outpatient services, skilled nursing facilities, ambulatory surgery centers, and urgent care clinics, there were moderate and significant differences among groups. Future investigation should analyze the impact of medical and social care integration on specific medical care service types.

The relative savings of addressing social needs supports the Health*Connections* program design, whereby anyone can contact the call center for a referral, negating the need to proactively stratify patients by risk levels. However, the subgroup analysis reveals the potential utility of targeting high-risk patient populations for social service interventions, as shown in previous research.^32,38^ These findings show a noteworthy $3134 (8.6%) relative reduction between study groups point estimates in the second year, although of limited significance (*P* = 0.10). Proactive identification of high-risk patients with potential unmet social needs through health assessments and predictive algorithms, as seen in other social service coordination programs,^11,39^ may be worthwhile to organizations seeking to reduce costs by addressing social needs.

In the sensitivity analysis, participants with *at least 1* social need met were compared to those whose needs were not met. This analysis revealed that although there was a 7% relative reduction in expenditures in the 12 months after the index referral date between groups, the result was not significant. It is possible that participants with at least some unmet needs may experience exacerbations of current health conditions because of unmet social needs, despite getting other social needs met.

### Limitations

Several caveats apply to these findings. First, the findings relate to a sample of participants who self-identified their social service needs and were motivated to call the referral hotline. In addition, subjects were categorized into groups using self-reports about whether or not their social needs were met. The participants who reported all of their social needs met may have been more diligent about managing their health care expenses as well as following up on their social services referrals. Although the degree to which this motivation to address social needs or report social needs met impacted expenditures is unknown, caution should be taken in generalizing these results to a general managed care population. Future research could evaluate the impact of addressing social determinants of health on medical expenditures through an experimental design in which participants are randomly assigned to either the usual social service referral program or to an enhanced social service navigation aimed at assuring and independently confirming access to social services.

Although MCO staff followed up with all participants to track whether or not the social service met their needs, a noteworthy number of program participants (n = 18,454) had not been contacted and, therefore, were excluded from these analyses. Participants were not contacted for a variety of reasons, including inability to reach them and those referred too soon to qualify for follow-up. It is not known whether these participants were more likely to report their social needs met or to utilize additional health care services. Despite this limitation, the sample size of each of the study groups was large enough for valid analysis. Improved patient engagement and follow-up would improve future program evaluation but incur additional program costs.^40^

It is unclear why the group who reported their needs were unmet had lower health care costs in the year before and after the social service referral. Although the models adjusted for disease severity and other observable factors, they did not account for potentially relevant unobserved covariates, such as education attainment, attitudes, social support, income level, support from other community programs, or functional disability, that may impact both medical and social service utilization. It is possible that unmeasured barriers negatively affect both health service and social service utilization. Alternatively, it may be that the group who reported all their social needs met has an unmeasured need for more health care that leads them to be more likely to obtain social services.

The social service tracking database did not record the reasons why participants report that their needs were not met. It is possible that certain participants were more likely to perceive social services negatively, or to perceive that the service was not of sufficient quality, or did not access the service at all. Furthermore, social service organizations outside the program may have served participants' social needs, a circumstance that would reduce the measured effect of the Health*Connections* program. The lack of more detailed tracking data may introduce potential selection bias issues. It is unclear how these may influence the direction of this observed effect or whether they effect the groups equally.

Lastly, collecting reliable data on the cost of social services from the community-based organizations, such as food pantries and homeless shelters, creates challenges to evaluating how social service needs affects total medical care expenditures. As such, it is not known whether savings realized by the MCO represents savings to the entire system. For organizations to fully incorporate social services into their health care delivery or insurance benefit structures, it may be necessary to validly and reliably define the social services and associated costs. Unfortunately, no comprehensive coding system exists that would enable providers to specify the social services delivered. A small number of billing codes (eg, home-delivered meals [S5170]) are included in the Healthcare Common Procedure Coding System developed by the Centers for Medicare & Medicaid Services.^41^ However, a more complete social services billing code set would permit more precise measurement, payment, and evaluation of social service care.^42,43^

## Conclusions

The objective of this study was to evaluate an MCO's call center-based social services coordination program to understand the relationship of social services to health care expenditures. This study found that by connecting participants with needed social services, the MCO significantly reduced health care expenditures. The MCO conceived Health*Connections* in recognition of the Institute of Medicine's call to improve coordination of the social and medical care systems by promoting community-based partnerships.^44^ These multi-sector collaborative partnerships hold promise for improving population health.^1^ Yet, financial incentives contribute only part of the potential benefits to social and medical care integration. Future research should examine how investments in social service interventions can improve community health outcomes.
